# Determining the Intellectual Structure and Academic Trends of Smart Home Health Care Research: Coword and Topic Analyses

**DOI:** 10.2196/19625

**Published:** 2021-01-21

**Authors:** Hyo-Jin Kang, Jieun Han, Gyu Hyun Kwon

**Affiliations:** 1 Department of Service Design Engineering Sungshin Women's University Seoul Republic of Korea; 2 Graduate School of Technology and Innovation Management Hanyang University Seoul Republic of Korea

**Keywords:** smart home, smart home health care, coword analysis, topic analysis, intellectual structure, academic trends

## Abstract

**Background:**

With the rapid development of information and communication technologies, smart homes are being investigated as effective solutions for home health care. The increasing academic attention on smart home health care has primarily been on the development and application of smart home technologies. However, comprehensive studies examining the general landscape of diverse research areas for smart home health care are still lacking.

**Objective:**

This study aims to determine the intellectual structure of smart home health care in a time series by conducting a coword analysis and topic analysis. Specifically, it investigates (1) the intellectual basis of smart home health care through overall academic status, (2) the intellectual foci through influential keywords and their evolutions, and (3) intellectual trends through primary topics and their evolutions.

**Methods:**

Analyses were conducted in 5 steps: (1) data retrieval from article databases (Web of Science, Scopus, and PubMed) and the initial dataset preparation of 6080 abstracts from the year 2000 to the first half of 2019; (2) data preprocessing and refinement extraction of 25,563 words; (3) a descriptive analysis of the overall academic status and period division (ie, 4 stages of 3-year blocks); (4) coword analysis based on word co-occurrence networks for the intellectual foci; and (5) topic analysis for the intellectual trends based on latent Dirichlet allocation (LDA) topic modeling, word-topic networks, and researcher workshops.

**Results:**

First, regarding the intellectual basis of smart home health care, recent academic interest and predominant journals and research domains were verified. Second, to determine the intellectual foci, primary keywords were identified and classified according to the degree of their centrality values. Third, 5 themes pertaining to the topic evolution emerged: (1) the diversification of smart home health care research topics; (2) the shift from technology-oriented research to technological convergence research; (3) the expansion of application areas and system functionality of smart home health care; (4) the increased focus on system usability, such as service design and experiences; and (5) the recent adaptation of the latest technologies in health care. Based on these findings, the pattern of technology diffusion in smart home health care research was determined as the adaptation of technologies, the proliferation of application areas, and an extension into system design and service experiences.

**Conclusions:**

The research findings provide academic and practical value in 3 aspects. First, they promote a comprehensive understanding of the smart home health care domain by identifying its multifaceted intellectual structure in a time series. Second, they can help clinicians discern the development and dispersion level of their respective disciplines. Third, the pattern of technology diffusion in smart home health care could help scholars comprehend current and future research trends and identify research opportunities based on upcoming research waves of newly adapted technologies in smart home health care.

## Introduction

The rapid development of information and communication technologies, including the Internet of Things (IoT) and ubiquitous computing, have made home environments highly intelligent and allow smart homes to be realized [1]. In previous research, the smart home was characterized as the integration of technologies such as home automation, automatically controlled systems, communication networks and connecting devices and services, remote access and control, and home intelligence, with the context awareness of users [2,3]. Accordingly, the purpose of smart homes has been defined as providing a better home life experience with enhanced security, safety, communication, comfort, and entertainment through technical management of the home environment [4]. On the other hand, ethical and legal concerns surrounding smart home technologies have been pointed out regarding the security, privacy, and confidentiality of users [5,6]. Nonetheless, with a prudent approach to these ethical and legal challenges, smart homes could be an effective tool for continuous, remote, and nonintrusive health monitoring and disease prevention while guaranteeing users’ independence and quality of life [7,8].

Various terms have been used to describe such technology, such as “smart homes in or for health care” [7,8], “health smart home” [9,10], and “ubiquitous health care” [11,12]. For consistent terminology, we adopted the term “smart home health care” and established the following operationalized definition [13], which embraces both technical and experiential perspectives:

Smart home health care is a health care service in one’s residence incorporated with IoT technology and ubiquitous computing, which has the characteristics of home automation and home intelligence, communication networks, and remote access and control by authorized health care personnel. It offers informal health care services such as real-time or long-term health monitoring, unobtrusive activity support without interference with daily lives, and disease prevention through anomaly detection. It can reduce care costs, allow satisfactory service experience in a comfortable and private home environment, and ensure the independence of residents.

The academic focus of previous smart home research has mainly been on the development and application of smart home technologies in the fields of computer science and engineering [2-4,14]. Likewise, smart homes for health care have primarily been examined from the perspective of technological application [7,8,15,16]. Such studies have analyzed and classified smart home health care services according to the type of sensor, network or communication technology, and algorithm models of data processing [3,7,8]. Despite the recent increase of technology-related research on smart home health care, comprehensive studies on the general state of its diverse research areas remain lacking.

Therefore, it is necessary to comprehend the current landscape of smart home health care research and seek future research opportunities. The specific research questions we used to investigate the intellectual structure of smart home health care are the following: (RQ1) What is the overall academic status of the current research on smart home health care, and which research fields have mainly been focused on? (RQ2) What are the representative keywords in the research on smart home health care, and how have they evolved over time? (RQ3) What are the intellectual trends in smart home health care research, and what are the main research orientations?

Thus, the objective of this study is to determine representative research topics on smart home health care and their evolutionary trends, and determine the intellectual structure of smart home health care research by conducting bibliometric network analysis. The resulting findings could lead to a comprehensive understanding of the current literature on smart home health care and enable scholars to extend their academic interests in future research.

## Methods

### Bibliometric Network Analysis and Coword Analysis

Bibliometric network analysis refers to a computer-assisted scientific review methodology that provides quantitative and statistical analysis by summarizing a large number of research publications through various descriptors and indicators [17]. Havemann and Scharnhorst [18] organized the approaches of bibliometric networks into the following categories: (1) citation networks of articles and journals; (2) bibliographic coupling networks; (3) cocitation networks of articles, authors, and journals; (4) co-authorship networks; and (5) word co-occurrence networks (ie, coword analysis). Among them, coword analysis has been considered an effective approach for understanding key topics in a certain research area, calculating the association strength of representative terms, and illustrating the field's knowledge structure by revealing patterns and trends among those topics [19,20]. Researchers have combined coword analysis with cluster analysis, multidimensional scaling, or social network analysis (SNA) to statistically investigate word co-occurrence patterns using titles, keywords, and abstracts [21,22]. In health informatics research, the combination of coword analysis and SNA has been adopted to identify the trends of specific themes in health care, such as mobile health [23], cybersecurity [24], and robotic or mixed reality surgery [25,26]. Accordingly, we adopted coword analysis combined with SNA to better understand key topics in smart home health care research. The analysis flow in this study was adopted from previous research [27,28] and included data retrieval, data preprocessing and refinement, and data analysis.

### Topic Analysis Combining Topic Modeling and Social Network Analysis

To complement the coword analysis with word co-occurrence networks, we adopted topic modeling to identify more detailed topic groups and their evolutionary trends. In machine learning and natural language processing, topic modeling is a prominent technique for data mining, latent data detection, and finding associations among data and text documents to discover hidden semantic structures [29,30]. Latent Dirichlet allocation (LDA) [31], one of the most popular text mining methods [32], was adopted in this study to identify latent topics in the retrieved data and classify words and documents into topics. We also combined topic modeling with SNA to establish word-topic networks and better understand the relations among topics.

### Data Collection and Analysis Process

#### Data Retrieval

We collected articles relevant to this research from the journal databases of Web of Science, Scopus, and PubMed. Web of Science and Scopus are two of the most authoritative scholarship databases, with peer-reviewed papers covering a wide range of subjects in various disciplines [33]. PubMed was also chosen in order to supplement domain-specific knowledge pertaining to health care. To retrieve the articles, a keyword search query was composed as follows:

***Query*** = ((“intelligent” OR “smart”) AND “home”) AND ((“health” AND “care”) OR “ehealth” OR “mhealth” OR “uhealth”))

Article types were limited to journal articles and proceeding papers, article language was constrained to English, and the time period was limited to the year 2000 onwards, considering the recency of smart home technologies. The data retrieval was conducted on July 1, 2019, and 6579 articles were initially collected (4683 from Web of Science, 1421 from Scopus, and 475 from PubMed). From the raw dataset, we excluded data that were duplicated among databases or that had no information on the year of publication, author, or abstract. The initial dataset of 6080 articles was prepared using the following bibliometric data: title, year of publication, author, journal, abstract, and keywords.

#### Data Preprocessing and Refinement

Noun data needed to be extracted from the abstract dataset in order to constitute a text corpus. For this, we conducted data preprocessing according to the stages of dictionary development: exception, definition, and synonyms. First, the “exception” dictionary was established to remove any general terminologies which are routinely utilized in abstracts to explain research structures, processes, and methodologies (eg, background, method, result, discussion, literature, purpose, implementation). Second, the “definition” dictionary was developed to define compound nouns as 1 word (eg, “smart home,” and not “smart” and “home” separately). To expand the definition dictionary, academic compound nouns were added to reflect the author keywords data. Third, the “synonym” dictionary was produced to detect duplicate meanings and replace them with 1 representative word (eg, “smart home” would represent “smart house,” “intelligent home,” “intelligent smart home,” and the plural forms of the same terms). The synonym dictionary also combined the singular and plural forms of the same terms into singular words. After this process, 25,563 words from 6080 abstracts were prepared as the text corpus for the following steps.

After defining the text corpus, data refinement was conducted in order to filter terms of high frequency but general usage. The dataset was arranged as a word-document matrix by occurrence, and was transformed into a co-occurrence matrix using the symmetric Ochiai-Salton algorithm. To filter normally used terminology, this study adopted the term frequency–inverse document frequency (TF-IDF) text-mining method. For data preprocessing and refinement, we utilized Python (version 3.7; Python Software Foundation [34]) and NetMiner (version 4.3; Cyram Inc [35]) commercial software. Since NetMiner software specializes in conducting semantic network analysis and offers a graphical demonstration of the network, it was also used in the following analysis stage to determine the networks and visualizations.

#### Data Analysis

**Figure 1:**
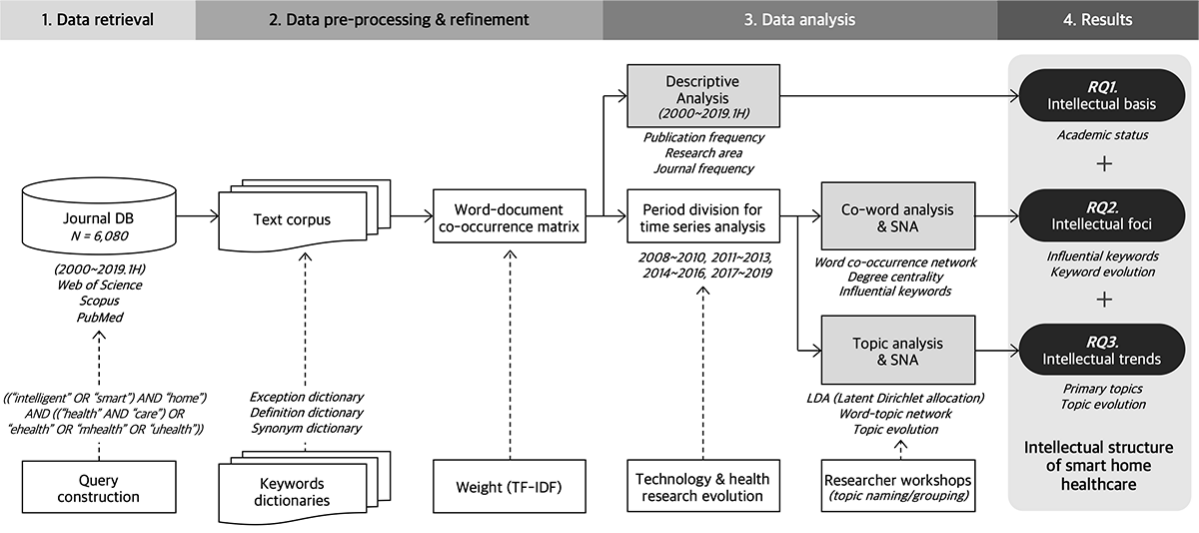
Research methodology. DB: database; RQ: research question; SNA: social network analysis; TF-IDF: term frequency–inverse document frequency.

Figure 1 depicts the overall process of this research methodology. The data analysis was conducted according to 3 subanalysis stages.

First, we conducted a descriptive analysis based on the frequency of publication. From this, the research areas and principal journals relevant to smart home health care research could be identified. The results from this analysis could answer the first research question regarding overall academic status as an intellectual basis for smart home health care. After the descriptive analysis, time periods were divided by a certain scale in order to conduct further time-series analyses and explore various evolutions. To decide the time scale, we examined the evolution of technology and health research, or how health care research has evolved in response to recent emerging technologies. For this, we searched for several keywords directly pertinent to the study and emerging technology keywords [36] (eg, artificial intelligence [AI], IoT, and blockchain) associated with health care on the Web of Science database. Considering the evolution of yearly publication frequencies for those keywords, the time scale was divided into 4 stages of 3-year blocks: 2008-2010, 2011-2013, 2014-2016, and 2017-2019.

Second, we conducted a coword analysis combined with SNA to establish word co-occurrence networks. This was based on document frequency, TF-IDF values, and the degree centrality of keywords in word co-occurrence networks. Influential keywords and keyword evolution could then be determined, answering the second research question regarding the intellectual foci. We constructed word co-occurrence networks to transform the word-document networks into word-word networks, having weights of word co-occurrence frequency with a proximity measure of the correlation type inner product. Using degree-centrality analysis and link reduction, influential keywords were extracted by filtering the top 100-degree centrality words. Primary keywords (the top 40) were positioned according to their degree centrality values and compared within the time series to understand their evolution.

Third, we conducted a topic analysis combining topic modeling and SNA to establish word-topic networks. This allowed us to investigate the primary topics in smart home health care research and their evolutions within the time series, answering the third research question about intellectual trends. For topic modeling, we adopted an LDA with a Markov Chain Monte Carlo learning method to identify latent topics and classify words and documents into topics. Word-topic networks were then constructed and visualized according to allocation probability values. Three authors of this study held 3 consecutive researcher workshops to interpret the topic analysis results qualitatively to (1) name the individual topics from the results of LDA separately, (2) compare the name results of the 3 researchers and establish 1 set of topic names by reaching a consensus on any discrepancies, and (3) classify them into superordinate topic groups regarding the visualized word-topic networks. Lastly, the document frequencies allocated to the topic groups were counted and compared among different time periods to comprehend the topic evolution.

## Results

### Descriptive Analysis

#### Overall Academic Status

**Figure 2:**
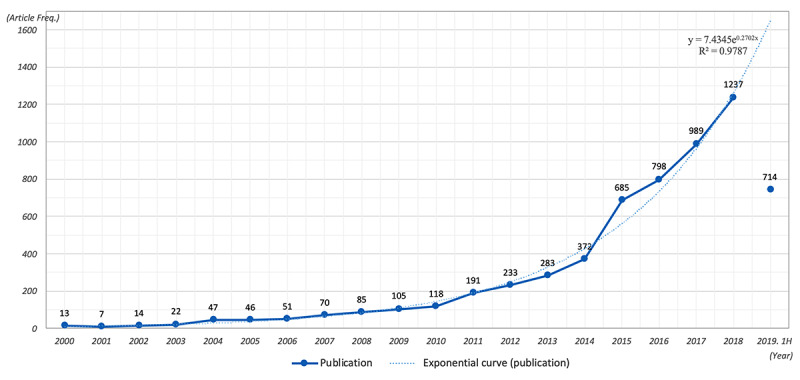
The evolution of publication frequencies from 2000-2019. (The publication frequency in 2019 was based on the first half of the year; thus, it was not included in calculating for the exponential curve.).

Using the initial 6080 articles, an overview of smart home health care research was compiled. Figure 2 shows the evolution of publication frequency from the year 2000, with the exponential curve being calculated to exclude the data on the first half of 2019. This shows that publication frequency rapidly increased in the last 10 years, illustrating the high level of research interest and academic popularity of smart home health care (*R*^2^=0.979).

Table 1 exhibits the list of significant journals in smart home health care research from the year 2000; the journal frequencies of 1548 journals were counted. The *Journal of Medical Internet Research* holds the highest rank with 504 publications, followed by *JMIR mHealth and uHealth*, *JMIR Research Protocols*, *International Journal of Medical Informatics*, *BMC Medical Informatics and Decision Making*, *BMC Public Health*, and *JMIR Mental Health*. The top 3 journals from JMIR Publications constituted 17.62% (1071 of 6080 articles) of the total articles on smart home health care research.

**Table 1:** The top influential journals in smart home health care research from 2000-2019.

Journal	Article frequency
Journal of Medical Internet Research	504
JMIR mHealth and uHealth	392
JMIR Research Protocols	175
International Journal of Medical Informatics	91
BMC Medical Informatics and Decision Making	77
BMC Public Health	65
JMIR Mental Health	65
Journal of Medical Systems	63
Trials	59
Plos One	54
Journal of Telemedicine and Telecare	51
Studies of Health Technologies and Informatics	48
Methods of Information in Medicine	46
Journal of the American Medical Informatics Association	44
Sensors	44
BMJ Open	43
Health Informatics Journal	41
IEEE Journal of Biomedical and Health Informatics	33
IEEE Access	32
International Journal of Environmental Research and Public Health	32
Translational Behavioral Medicine	32
Journal of Health Communication	31
Patient Education and Counseling	31
ACM International Conference Proceeding Series	29
BMC Health Services Research	28
Telemedicine and eHealth	26
Contemporary Clinical Trials	25
JMIR Cancer	23
Aids and Behavior	22
CIN: Computers, Informatics, Nursing	22
Global Health Action	22
Journal of Biomedical Informatics	22

**Figure 3:**
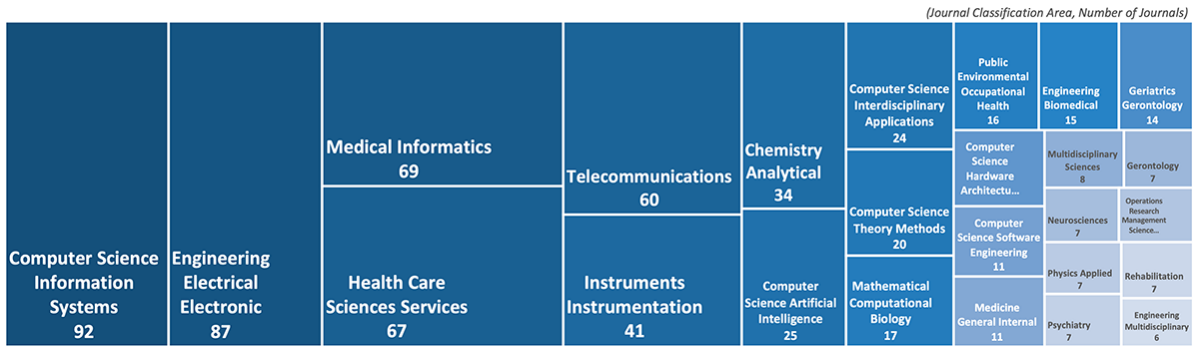
The academic area classification of journals on smart home health care research by Web of Science.

According to the academic area classification of Web of Science, the subject or type of journals that are pertinent to smart home health care research, derived with the same search query, is shown in Figure 3. The most predominant research domains are still from the engineering side, such as *Computer science information system* (23.77%) and *Electrical or electronic engineering* (22.48%). However, studies from medical and health care areas, such as *Medical informatics* (17.83%) and *Health care sciences and services* (17.31%), also constitute considerable portions of smart home health care research. Various medical fields, such as *Geriatrics or gerontology*, *Neurosciences*, *Rehabilitation*, and *Psychiatry,* emerged as small portions of the total research since advanced smart home health care technologies have already been heavily investigated in engineering domains.

#### Period Division in Association with Emerging Technologies

**Figure 4:**
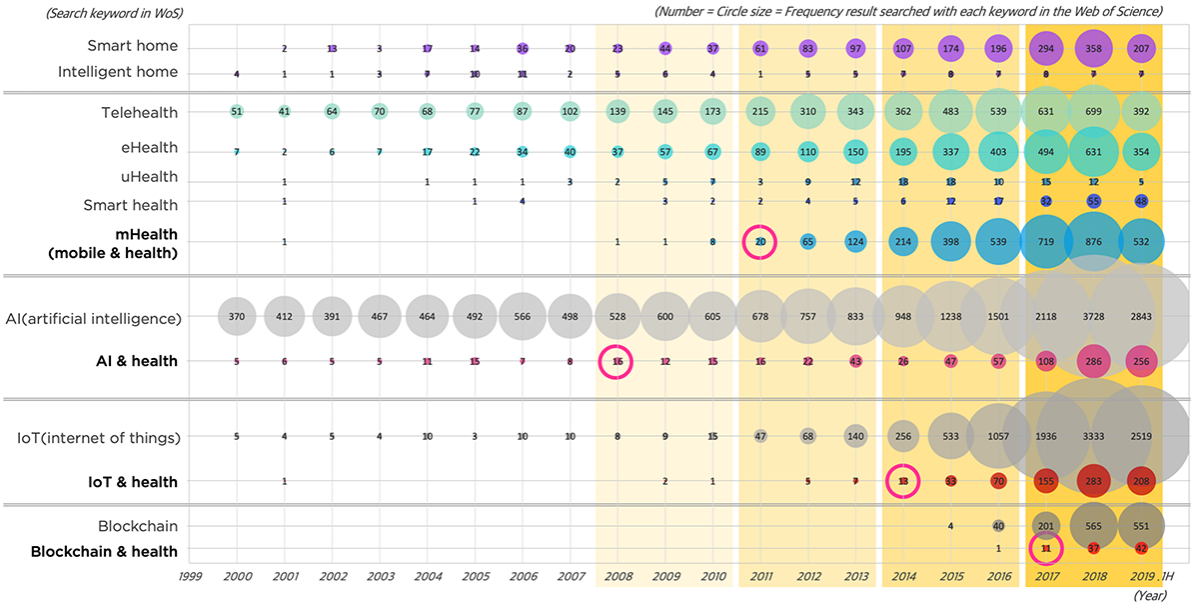
The evolution of publications relevant to emerging technologies and health research from 2000-2019.

As explained in the Methods section, the evolution of technology in association with health research was examined in order to select a time scale for further time-series analyses. The advent of new technologies can influence health care research and be adopted in health care and medical fields in diverse ways. Hence, several keywords used in the query of this study and emerging technology keywords [36] coupled with health care were searched in the Web of Science, and the publication frequencies were counted yearly from the year 2000. The result is illustrated in Figure 4.

We specifically focused on the keywords “mHealth” (ie, mobile health), “AI and health,” “IoT and health,” and “blockchain and health,” which are technologies with significant influence on data collection and processing, service delivery, and interactions. The moments of advent were identified as the moments where publication frequency doubled compared to the previous year, exceeded 10 for the first time, and continued to gradually increase; this occurred for AI and health in 2008, mHealth in 2011, IoT and health in 2014, and blockchain and health in 2017. As AI had been greatly researched prior to 2000, the adaptation of AI in the health care field also occurred early, particularly in AI-assisted medical diagnosis research. Given this background, the advent year of the AI and health topic could be decided earlier than those of other topics. In accordance with the distribution of frequency, a time scale of 4 stages of 3-year blocks (2008-2010, 2011-2013, 2014-2016, and 2017-2019) was chosen. Despite the existence of telehealth research in the years between 2000 and 2007, this initial period was not included in the time-series analysis because the number of articles published at the time was insufficient to conduct a bibliometric network analysis, as shown in Figure 2.

#### Coword Analysis for the Intellectual Foci

In accordance with the time scale division, Table 2 shows general information on the words and documents per period. Over the total period, 25,556 words were extracted from 5810 documents. Approximately 75.0% of keywords occurred in 1-3 documents, 10.2% in 4-7 documents, and 5.0% in 8-13 documents. On average, any given keyword appeared in 9.2 documents, with the actual number of documents in which a given keyword appeared ranging from 1 to 813. The number of documents drastically increased throughout 4 periods, and period 4 contained half of the total documents (50.6%) despite the data in 2019 only comprising half a year. The number of words has also considerably grown, with the words from period 4 occupying 65.9% of the total 25,556 words.

Both the number of links created from word-document networks and those calculated by TF-IDF (which can represent how significant a word is in a certain document by eliminating ordinary words with high frequency but low importance) constituted more than half of the total networks. The evolution of documents and keywords could also imply that smart home health care research has been rapidly developing quantitatively and qualitatively.

**Table 2:** General information of the time-series analysis.

Variables	Total period (2008-2019)	Period 1 (2008-2010)	Period 2 (2011-2013)	Period 3 (2014-2016)	Period 4 (2017-2019)
Number of documents, n (%)	5810 (100)	308 (5.3)	707 (12.2)	1855 (31.9)	2940 (50.6)
Number of words, n (%)	25,556 (100)	3814 (14.9)	6868 (26.9)	12,749 (49.9)	16,831 (65.9)
Number of links in the word-document network, n (%)	333,921 (100)	14,724 (4.4)	36,642 (11.0)	107,405 (32.2)	175,150 (52.5)
Number of links in the word-document network, TF-IDF (%)	223,422 (100)	10,643 (4.8)	25,342 (11.3)	72,230 (32.3)	115,207 (51.6)

Among the word-document networks, the links whose TF-IDF values were above 0.5 and the top 10% of words in terms of document frequency were extracted. Then, word co-occurrence networks were also constructed in order to determine the primary keywords based on precise figures, namely degree centrality. Since the links from the original networks were too massive (as shown in Table 3), they were extracted in 2 steps: (1) by the top 100 words in degree centrality values, and (2) by link reduction according to a threshold of word co-occurrence frequency. “Network degree centralization“ is a measurement that assesses the degree of inequality compared with a perfect “star network,” which is the most unequal network type [37]. The values of the network degree centralization index changed during the time series, showing a relative decentralization in periods 2 and 4. This may indicate a horizontal expansion of research toward the significant subject areas during those periods.

**Table 3:** Properties of word co-occurrence networks by time series.

Properties		Period 1 (2008-2010)	Period 2 (2011-2013)	Period 3 (2014-2016)	Period 4 (2017-2019)
**Word co-occurrence network information, n**					
	Links in the original network	184,550	414,721	1,084,449	1,576,645
	Links in the extracted network 1 (by the top 100 words in degree centrality values)	4053	4705	4943	4931
	Links in the extracted network 2 (by link reduction)	236	302	318	380
	Threshold of co-occurrence frequencies for link reduction^a^	7	10	25	12
Network degree centralization, index %		30.2	23.7	47.1	34.4
**Distribution of degree centrality**					
	Mean of degree centrality	0.048	0.061	0.064	0.077
	Standard deviation	0.074	0.072	0.079	0.077
	Min. of degree centrality	0.000	0.000	0.000	0.000
	Max. of degree centrality	0.343	0.293	0.525	0.414

^a^The threshold value was decided by a link reduction simulation, identifying the point where the network component number suddenly increased.

Moreover, the evolution of word co-occurrence networks was visualized using document frequency as the node size for each word, as exhibited in Multimedia Appendix 1. In period 1, the keywords “monitoring,” “smart home,” “communication,” “home care,” and “environment” were highlighted, while most of the remaining keywords were not sufficiently distinguished. In period 2, the top keywords in period 1 gained more prominence, and several additional words (eg, “sensor,” “activity,” and “work”) noticeably emerged. In period 3, mHealth-relevant keywords, such as “mobile phone” and “SMS,” became distinct. In period 4, existing keywords (eg, “mobile phone,” “mobile app,” “barrier,” “experience,” and “strategy”) expanded their links. In comparison to prior periods, particularly period 1, the emphasized keywords in period 4 depicted a relatively even distribution of weights (ie, size of nodes); this implies that various research topics had been generated and investigated by many studies.

To determine the influential keywords and keyword evolutions in response to the second research question on intellectual foci, 40 primary keywords were determined based on their degree centrality values from the word co-occurrence networks (as shown in Multimedia Appendix 2). The primary keywords were categorized into the different groups based on their degree centrality values: core, semiperiphery, and periphery. The centrality value thresholds were decided based on the number of keywords in each group according to the data on degree centrality [38]. “Monitoring and (probably remote) communication in smart homes for home care of the elderly” was the main theme combining the core keywords in period 1. In period 2, the centrality values of “smart home,” “activity,” and “environment” increased in the core group, while “sensor” moved from the semiperiphery group to the core group. It may be inferred that research on smart home health care technologies, such as cases utilizing various types of sensors, was heavily conducted in this stage. In period 3, with the proliferation of mHealth research, keywords such as “mobile phone” and “SMS” shifted from the semiperiphery or periphery group to the core group. In period 4, “mobile app” transferred from the semiperiphery to the core group, while “barrier” and “experience” leaped from the periphery to the core group. This may imply that the applicability and user experience (eg, engagement, acceptability) of smart home health care services gained strong research attention in this period. The primary keywords extracted from the centrality analysis showed consistent results with the highlighted keywords in the previous word co-occurrence networks; this may enhance the validity of the deductive process in determining intellectual keywords and their changes.

### Topic Analysis for the Intellectual Trends

**Figure 5:**
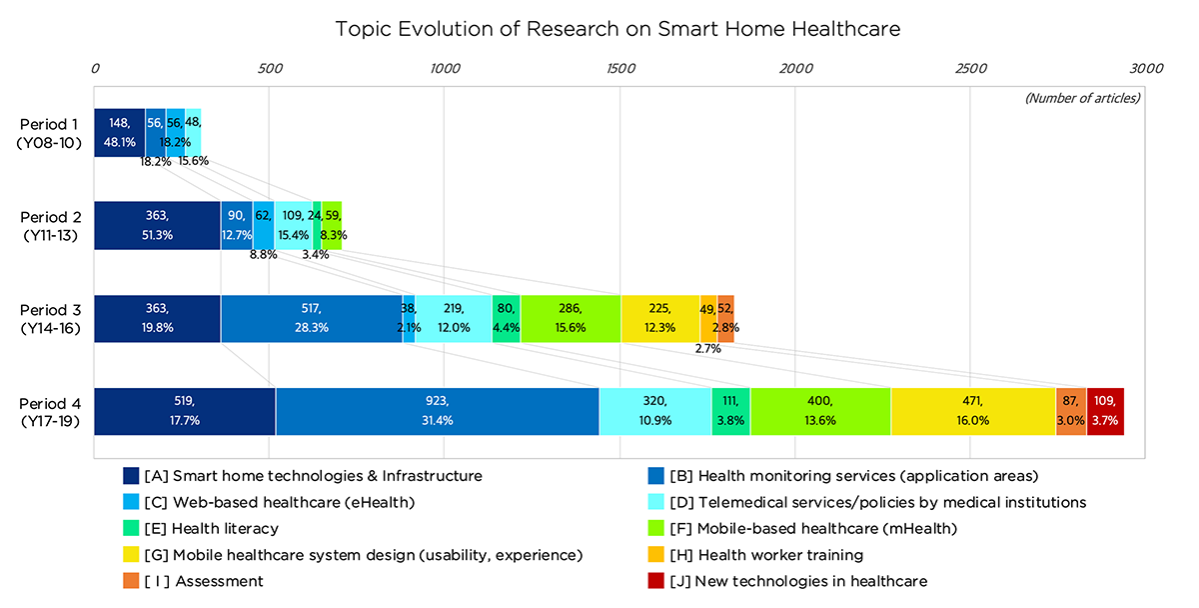
Topic evolution of research on smart home health care.

As explained in the Methods section, a topic analysis combining topic modeling and SNA was conducted in order to investigate the primary research topics on smart home health care and their evolutions; this would address the third research question on intellectual trends. During the LDA topic modeling process, the number of topics were decided based on the number of documents in each period (ie, 13 topics in period 1, 20 topics in period 2, and 30 topics in both periods 3 and 4). According to the allocation probability of a word per topic, word-topic networks were constructed and visualized, extracting the top 100 to 150 words in probability values for effective plotting. During the 3 researcher workshop sessions, we established the name of each topic, embracing the top 5 keywords of allocation probability individually in the first session and together in the second, in order to arrive at a consensus regarding any discrepancies. In the last session, we classified the topics into groups, considering their meanings and network dispositions from the visualized word-topic networks. Figure 5 illustrates the topic evolution, showing the frequencies and percentages of topics in each period; Table 4 displays the topic analysis results of topic naming and grouping; and Multimedia Appendix 3 visualizes the word-topic networks associated with topic groups.

Through the analysis of topic modeling and word-topic networks, the dynamics of topic evolution were used to identify the intellectual trends regarding smart home health care. These trends can be summarized in 5 aspects: (1) the diversification of smart home health care research topics; (2) the shift from technology-oriented research to technological convergence research; (3) the expansion of application areas and system functionality of smart home health care; (4) the increased focus on system usability, such as service design and experiences; and (5) the recent adaptation of the latest technologies in health care.

**Table 4:** Topic analysis results from (1) LDA topic modeling, (2) word-topic network, (3) topic naming and grouping workshops.

Period and topic group		Topic number	Topic name	Document frequency
**Period 1**				
	**A**	T03	Telemonitoring	20
		T04	Monitoring technology/infrastructure	24
		T05	IT-based decision support	18
		T09	Smart home activity detection	59
		T10	eHealth infrastructure	27
	**B**	T01	Lifetime family care	15
		T06	Elderly care	13
		T08	Robot-based health service	15
		T13	Health event management	13
	**C**	T07	Web-based health education	21
		T11	Web-based health contents delivery	35
	**D**	T02	Security architecture in hospitals	27
		T12	Care delivery strategy	21
**Period 2**				
	**A**	T01	Elderly care	66
		T02	Network integration	30
		T04	Signal/image detection	31
		T09	Communication platform	37
		T12	Privacy and security	40
		T13	Classification algorithm	25
		T14	Smart home activity detection	79
		T15	Monitoring technology/infrastructure	55
	**B**	T03	Chronological disease	26
		T05	Clinical service	20
		T06	PHR based on social media	22
		T20	Health activity management	22
	**C**	T07	Online patient community	41
		T17	Age-sensitive web accessibility	21
	**D**	T08	Telemedical service	33
		T11	Primary care delivery	26
		T19	Care delivery policy	50
	**E**	T10	eHealth literacy	24
	**F**	T16	Healthcare ICT investment (mobile)	38
		T18	Mobile-based health contents delivery	21
**Period 3**				
	**A**	T01	IoT and sensor network system	146
		T04	Activity sensing algorithm	126
		T13	Telemonitoring infrastructure	44
		T18	Privacy and security	47
	**B**	T06	e-Fitness	24
		T08	Chronological disease	46
		T12	Self-management	54
		T15	Exercise management	106
		T16	Elderly care	105
		T20	Cancer management	45
		T22	Mental health management	58
		T26	Lifetime family care by gamification	29
		T27	Chronological disease management	50
	**C**	T11	eHealth service benefit	38
	**D**	T07	Telemedical service	40
		T28	Care delivery policy	179
	**E**	T24	Health literacy	80
	**F**	T02	Health behavior awareness (mobile)	24
		T10	Age/gender-sensitive notification	58
		T17	Medication management (mobile)	80
		T25	Community healthcare (mobile)	72
		T29	mHealth data communication	52
	**G**	T05	mHealth technology acceptance	30
		T14	eHealth system design	63
		T21	mHealth app design	56
		T23	Usability for health workers/patients	49
		T30	System design	27
	**H**	T09	Health worker training	49
	**I**	T03	Assessment accuracy	52
	N/A^a^	T19	Etc	26
**Period 4**				
	**A**	T19	Activity sensing algorithm	212
		T20	Smart home technology infrastructure	307
	**B**	T03	Activity management	120
		T12	Chronological disease management	67
		T13	Mental healthcare	73
		T17	Emergency management	72
		T18	Rehabilitation training	70
		T21	Cancer management	89
		T22	Mental healthcare	130
		T24	Chronological disease management	56
		T25	Telemonitoring	71
		T26	Weight management	95
		T28	Age/gender-sensitive service	80
	**C**	N/A	N/A	N/A
	**D**	T02	Care cost-effectiveness (hospital)	61
		T05	Telemedicine platform	78
		T10	Community service	181
	**E**	T04	Health literacy	111
	**F**	T01	Mobile support for smoking cessation	109
		T07	Lifetime family care (mobile)	88
		T15	Healthcare service providing (mobile)	117
		T23	Medication management (mobile)	86
	**G**	T06	mHealth system design	160
		T14	Health system design	37
		T16	Elderly technology acceptance	69
		T27	Medical healthcare app	65
		T29	Technology acceptance	75
		T30	Mobile health system design	65
	**H**	N/A	N/A	N/A
	**I**	T11	Assessment algorithm	87
	**J**	T08	Blockchain and healthcare	66
		T09	Telemedicine/telesurgery technology	43

^a^N/A: not applicable.

The research topics on smart home health care were diverse throughout the 4 time periods. Based on the preliminary topics from LDA modeling, 10 topic groups were identified: (A) smart home technologies and infrastructure, (B) health monitoring services (application areas), (C) web-based health care (eHealth), (D) telemedical services and policies by medical institutions, (E) health literacy, (F) mobile-based health care (mHealth), (G) mobile health care system design (usability, experience), (H) health worker training, (I) assessment, and (J) new technologies in health care

It was found that the number of topic groups present increased in every period. As shown in Table 4, period 1 started with 4 topic groups, namely, “smart home technologies and infrastructure,” “application areas of health monitoring services,” “web-based health care (eHealth),” and “telemedical services and policies by medical institutions.” In period 2, 2 topic groups—“health literacy” and “mobile-based health care (mHealth)”—emerged. In period 3, another 3 topic groups were added: “mobile health care system design,” “health worker training,” and “assessment.” Notably, in period 4, one topic group, “new technologies in health care,” was added, and 2 topic groups, “web-based health care (eHealth)” and “health worker training,” were withdrawn. Though the topic group on eHealth decreased continuously from period 1, this does not mean that research on eHealth simply vanished; the relevance of eHealth to smart home health care could have simply reduced when compared to that of mHealth, as represented by topic groups (F) and (G).

Second, results showed that the majority of the research focus gradually shifted from technology-oriented research to technological convergence research. In Table 4, in topic group A, diverse smart home health care technology-oriented research topics were investigated, such as “smart home activity detection” (P1-T09, P2-T14), “monitoring technology/infrastructure” (P1-T04, P2-T15), “privacy and security issues in smart home for health care technology” (P2-T12, P3-T18), “IoT and sensor network system” (P3-T01), “activity sensing algorithm” (P3-T04, P4-T19), and “smart home technology or infrastructure (P4-T20).” The portion of this topic group constituted approximately 50% of all articles in periods 1 and 2, but it shrank to below 20% in periods 3 and 4, even though their frequencies slightly increased. Instead, technological convergence was enlarged in smart home health care research, investigating diverse application areas of smart home health care services, as explained in the next aspect.

Third, the application areas and functionality of smart home health care expanded as topic group B (Table 4) attained the highest frequency in periods 3 and 4. The subtopics in this group showed diverse areas of smart home health care, such as “mental health management” (P3-T22, P4-T13/T22), “chronological disease management” (P2-T03, P3-T08/T27, P4-T12/T24), “activity management” (P2-T20, P4-T03), “cancer management” (P3-T20, P4-T21), “elderly care” (P1-T06, P3-T16), “exercise management” (P3-T15), “weight management” (P4-T26), “emergency management” (P4-T17), and “rehabilitation training” (P4-T18), in their order of frequency. Moreover, in the early periods, the application areas were more focused on extended medical care, such as the management of elderly care and chronological disease, or “clinical services” (P2-T05). However, in the later periods, diverse health care services in daily lives, such as “lifetime care by gamification” (P3-T26) and “e-fitness” (P3-T06), emerged.

Fourth, the focus on system usability, such as service design experiences, increased from period 3 (as demonstrated by topic group G, Table 4). The major subtopics with high frequencies in this group were the aspects of system or application design issues, such as “mHealth system design” (P4-T06, T30), “mHealth app design” (P3-T21, P4-T27), and “eHealth system design” (P3-T14). In addition, the user aspects of service experiences were also accentuated in periods 3 and 4, as shown by “technology acceptance” (P4-T29), in association with the unified theory of acceptance and use of technology [39,40], and “usability for health workers or patients” (P3-T23) [41,42].

Lastly, the results showed that the latest technologies were being adapted in smart home health care research, as seen in topic group J (Table 4), which emerged in period 4. The subtopics of this group were “blockchain and health care” (P4-T08) and “telemedicine or telesurgery technology” (P4-T09). As illustrated in Figure 4, blockchain technology emerged just before period 4, and research on the topic coupled with health care began to increase in association with data privacy and security [43,44] and system interoperability [45]. Furthermore, the role of telemedicine and smart home health care services in surgery domains, as part of perioperative or postoperative procedures, gained more academic interest during this period [46].

## Discussion

### Principal Findings

Based on the analysis results, several discussion points emerged regarding the intellectual structure of smart home health care research.

First, mobile technologies have broadened the scope of private spaces and accelerated the expansion of home care to smart health care services. In the past, personal health care was associated with the home environment, but the intervention of mobile or smartphones has expanded the boundary of personal health care beyond the home. Accordingly, the adoption of mobile technologies in smart home services has increased and diversified the service areas, service functions, usability, and experiences of home care services. As shown in Multimedia Appendix 2, “mobile phone” and “mobile app” emerged in the core group in periods 3 and 4, instead of “smart home,” which was in the core in periods 1 and 2. This may imply that the home concept includes mobile personal space with regard to health care.

Second, smart technology evolution has enabled hospital-led services to be offered as personalized services. Hospital-led telehealth research in the past has been infused with home health care research, and the mobile technologies that are relevant to physiological monitoring and privacy protection have enhanced the association of hospital care and personal home care. While a large part of existing research in medical informatics used to focus on electronic medical records (EMR), recent research interest has been focused on personal health records (PHR) due to the adaptation of enabling mobile technologies (eg, IoT, sensor technology, blockchain) [47,48]. Moreover, mobile technologies could realize the concept of connected hospital or extended medical experiences, enhancing remote medical examination, treatment, and management.

Third, the pattern of technology innovation diffusion [49,50] in smart home health care research may be identified from the topic evolution results. Based on the findings in the topic evolution analysis, the technology diffusion in smart home health care fields has evolved from (1) the adaptation of technologies and (2) proliferation of application areas to (3) extension into system design and service experiences. Thus, it may be anticipated that the perspective of service design and experiences with diverse smart home health care application areas would be developed in the near future. Furthermore, when new technology appears in health care industries, a similar pattern of research evolution could be expected. For instance, blockchain technologies, which were recently adapted in smart home health care research, became an emerging topic (Table 4, P4-T08) in period 4. This technology could lead to a further investigation of the diverse application areas of smart home health care, resulting in a smart home health care system design coupled with blockchain technology and user experience features.

Lastly, this pattern of the technology diffusion process in smart home health care could have particular aspects caused by the medical or health care field's unique characteristics. Ethical and legal issues pertaining to personal health data are frequent concerns when technologies are integrated into health care services. Policies, regulations, and legislation systems relating to privacy and security issues in smart home health care services require greater attention than other commercial services [51,52]. Moreover, health care services usually demand a large number of clinical trials and verifications in order to generalize their applicability. Therefore, the technology diffusion process in smart home health care may take more prudence and time to evolve into further stages than other types of smart services. Nevertheless, as shown in Figure 4, the time lag of the adaptation of state-of-the-art technology in health care industries becomes shorter (eg, from AI to “AI and health,” from IoT to “IoT and health,” and from blockchain to “blockchain and health”). With the fourth industrial revolution, the cycle of technology diffusion in smart home health care could be gradually reduced.

### Limitations and Further Research

Despite the findings on the intellectual structure of smart home health care research, this study has several limitations due to its methodological approach. First, as coword analysis and topic analysis are quantitative and statistical analyses that summarize a large number of publications, qualitative research would be required in order to investigate specific topics in-depth among the various topic groups exhibited in Table 4.

Coword analysis is suggested as a prior stage to a systematic review to guide and accelerate the review process [53]. Therefore, a qualitative and thorough systematic literature review investigating specific topic groups can be conducted in future research. Second, this study adopted a semantic network analysis approach based on keywords and abstracts in order to identify keywords and topics in smart home health care research. However, the traditional approach in bibliometric analysis, which is based on co-authorship and citation information, could be applicable in future studies to investigate knowledge flows, research groups, and organizational influences. Third, this study extracted a text corpus only from noun data, following the general approach of semantic network analysis. However, if a text corpus is extracted from both adjectives and nouns, a more specific context in the usage of noun data may be explored with further analysis. Last, the text corpus was established from academic articles; thus, the perspective of commercial and business-wise cutting-edge technologies in the health care industry might not have been sufficiently included in this study’s findings.

### Conclusions

This research aimed to determine the representative research keywords and topics in smart home health care research and their evolutionary trends to demonstrate the intellectual structure of smart home health care by conducting bibliometric network analysis. First, we identified the academic status of the intellectual basis for smart home health care. With the recent increase in academic interest, smart home health care has been primarily investigated in research domains such as computer science information systems, electrical engineering, medical informatics, and health care science and services. Second, keywords and centrality analysis were conducted based on word co-occurrence networks to determine the intellectual foci. The evolution of primary keywords was demonstrated throughout 4 periods, and the top 3 keywords in each period were identified: “monitoring, communication, smart home,” “smart home, monitoring, sensor,” “mobile phone, SMS, monitoring,” and “mobile app, barrier, experience,” respectively. Third, we conducted a topic analysis based on topic modeling and word-topic networks in order to determine the intellectual trends. Accordingly, 5 aspects of topic evolution emerged: (1) the diversification of smart home health care research topics, (2) the shift from technology-oriented research to technological convergence research, (3) the expansion of application areas and system functionality of smart home health care, (4) the increased focus on system usability, such as service design and experiences, and (5) the recent adaptation of the latest technologies in health care.

Through these findings, this study discussed the role of mobile and smart technologies in relation to the expansion of home care to smart health care services, and the extension of hospital-led services to personalized services. Moreover, the pattern of technology diffusion in smart home health care research was verified as (1) the adaptation of technologies, (2) the proliferation of application areas, and then (3) the extension into system design and service experiences. This pattern may take more time to evolve due to the ethical and legal concerns surrounding personal health data; however, the cycle of technology diffusion in smart home health care is expected to shorten gradually.

The value and potential contributions of these findings can be summarized in 3 aspects. First, they promote a comprehensive understanding of the smart home health care domain by determining its intellectual structure in a time series. The results disclosed and structuralized the massive knowledge body of smart home health care, which has been investigated from diverse research domains but still lacked in comprehending the general landscape. Second, these findings can help clinicians recognize the development and dispersion level of their disciplines and specialty topics. Moreover, practitioners can recognize other related topics to their major disciplines and seek any cooperation opportunities associated with smart home health care services. Third, the pattern of technology diffusion in smart home health care can enable scholars to understand current and future research trends. Particularly, this study is valuable because it confirmed that the pattern of technology diffusion could be applicable in smart home health care; thus, researchers can anticipate the upcoming research waves of newly adapted technologies in smart home health care and explore further research opportunities. Moreover, as a practical implication, the technological trends and their diffusion patterns in smart home health care can be utilized when professionals investigate market status, analyze their competitive advantages, and establish business strategies in their respective practice areas.

## Appendix

Multimedia Appendix 1 Word co-occurrence network evolution of research on smart home health care.

Multimedia Appendix 2 The evolution of the position of the top 40 keywords by degree centrality in word co-occurrence networks.

Multimedia Appendix 3 Word-topic network evolution of research on smart home health care.

## Abbreviations

AI: artificial intelligence
eHealth: web-based health
IoT: Internet of Things
LDA: latent Dirichlet allocation
mHealth: mobile health
SNA: social network analysis
TD-IDF: term frequency–inverse document frequency
uHealth: ubiquitous health (or health services delivered using ubiquitous technology, such as radio frequency identification)

## References

[1] Bassi A, Horn G (2008). Internet of Things in 2020: Roadmap for the Future. European Commission: Information Society and Media.

[2] Park SH, Won SH, Lee JB, Kim SW (2003). Smart home: digitally engineered domestic life. Personal Ubiquitous Comput. (3-4).

[3] Alam MR, Reaz MBI, Ali MAM (2012). A Review of Smart Homes—Past, Present, and Future. IEEE Trans. Syst., Man, Cybern. C.

[4] Aldrich F, Harper R (2006). Smart homes: past, presentfuture. Inside the smart home.

[5] Pisani AR, Wyman PA, Mohr DC, Perrino T, Gallo C, Villamar J, Kendziora K, Howe GW, Sloboda Z, Brown CH (2016). Human Subjects Protection and Technology in Prevention Science: Selected Opportunities and Challenges. Prev Sci.

[6] Kelly P, Marshall SJ, Badland H, Kerr J, Oliver M, Doherty AR, Foster C (2013). An ethical framework for automated, wearable cameras in health behavior research. Am J Prev Med.

[7] Amiribesheli M, Benmansour A, Bouchachia A (2015). A review of smart homes in healthcare. J Ambient Intell Human Comput.

[8] Majumder S, Aghayi E, Noferesti M, Memarzadeh-Tehran H, Mondal T, Pang Z, Deen MJ (2017). Smart Homes for Elderly Healthcare-Recent Advances and Research Challenges. Sensors (Basel).

[9] Rialle V, Duchene F, Noury N, Bajolle L, Demongeot J (2002). Health "Smart" home: information technology for patients at home. Telemed J E Health.

[10] Bartolomeu P, Fonseca J, Vasques F (2008). Challenges in Health Smart Homes. https://ieeexplore.ieee.org/document/4571016.

[11] Deen MJ (2015). Information and communications technologies for elderly ubiquitous healthcare in a smart home. Pers Ubiquit Comput.

[12] Rahmani A, Thanigaivelan N, Tuan NG, Granados J, Negash B, Liljeberg P, Tenhunen H (2015). Smart e-Health Gateway: Bringing intelligence to Internet-of-Things based ubiquitous healthcare systems. https://ieeexplore.ieee.org/document/7158084.

[13] Kang H, Han J, Kwon GH (2019). An Ecological Approach to Smart Homes for Health Care Services: Conceptual Framework of a Smart Servicescape Wheel. JMIR Mhealth Uhealth.

[14] Saad al-sumaiti A, Ahmed MH, Salama MMA (2014). Smart Home Activities: A Literature Review. Electric Power Components and Systems.

[15] Chan M, Estève Daniel, Escriba C, Campo E (2008). A review of smart homes- present state and future challenges. Comput Methods Programs Biomed.

[16] Demiris G, Hensel BK (2018). Technologies for an Aging Society: A Systematic Review of “Smart Home” Applications. Yearb Med Inform.

[17] De Bellis N (2009). Bibliometrics and Citation Analysis: From the Science Citation Index to Cybermetrics.

[18] Havemann F, Scharnhorst A (2012). Bibliometric Networks.

[19] Lee WH (2008). How to identify emerging research fields using scientometrics: An example in the field of Information Security. Scientometrics.

[20] Leydesdorff L, Welbers K (2011). The semantic mapping of words and co-words in contexts. Journal of Informetrics.

[21] Hu J, Zhang Y (2015). Research patterns and trends of Recommendation System in China using co-word analysis. Information Processing & Management.

[22] Schrock WA, Zhao Y, Richards KA, Hughes DE, Amin MS (2018). On the nature of international sales and sales management research: a social network–analytic perspective. Journal of Personal Selling & Sales Management.

[23] Peng C, He M, Cutrona SL, Kiefe CI, Liu F, Wang Z (2020). Theme Trends and Knowledge Structure on Mobile Health Apps: Bibliometric Analysis. JMIR Mhealth Uhealth.

[24] Jalali MS, Razak S, Gordon W, Perakslis E, Madnick S (2019). Health Care and Cybersecurity: Bibliometric Analysis of the Literature. J Med Internet Res.

[25] Han J, Kang H, Kim M, Kwon GH (2020). Mapping the intellectual structure of research on surgery with mixed reality: Bibliometric network analysis (2000-2019). J Biomed Inform.

[26] Shen L, Wang S, Dai W, Zhang Z (2019). Detecting the Interdisciplinary Nature and Topic Hotspots of Robotics in Surgery: Social Network Analysis and Bibliometric Study. J Med Internet Res.

[27] Heradio R, de la Torre L, Galan D, Cabrerizo FJ, Herrera-Viedma E, Dormido S (2016). Virtual and remote labs in education: A bibliometric analysis. Computers & Education.

[28] Du HS, Ke X, Chu SK, Chan LT (2017). A bibliometric analysis of emergency management using information systems (2000-2016). OIR.

[29] Blei DM (2012). Probabilistic topic models. Commun. ACM.

[30] Deerwester S, Dumais ST, Furnas GW, Landauer TK, Harshman R (1990). Indexing by latent semantic analysis. J. Am. Soc. Inf. Sci.

[31] Blei D, Ng A, Jordan M (2003). Latent Dirichllocation Allocation. The Journal of Machine Learning Research.

[32] Jelodar H, Wang Y, Yuan C, Feng X, Jiang X, Li Y, Zhao L (2018). Latent Dirichlet allocation (LDA) and topic modeling: models, applications, a survey. Multimed Tools Appl.

[33] Meho LI, Yang K (2007). Impact of data sources on citation counts and rankings of LIS faculty: Web of science versus scopus and google scholar. J. Am. Soc. Inf. Sci.

[34] Guzdial M, Ericson B (2015). Introduction to Computing and Programming in Python, 4th Edition.

[35] Cyram Inc (2013). NetMiner. Social Network Analysis Software.

[36] Cearley D, Burke B, Searle S, Walker M (2017). Top 10 Strategic Technology Trends for 2018. GartnerTrends.

[37] Wasserman S, Faust K (1994). Social Network Analysis: Methods and Applications.

[38] Ronda-Pupo GA, Guerras-Martin L� (2011). Dynamics of the evolution of the strategy concept 1962-2008: a co-word analysis. Strat. Mgmt. J.

[39] Hoque R, Sorwar G (2017). Understanding factors influencing the adoption of mHealth by the elderly: An extension of the UTAUT model. Int J Med Inform.

[40] Tavares J, Oliveira T (2017). Electronic Health Record Portal Adoption: a cross country analysis. BMC Med Inform Decis Mak.

[41] Cruz Zapata B, Hernández Niñirola Antonio, Idri A, Fernández-Alemán José Luis, Toval A (2014). Mobile PHRs compliance with Android and iOS usability guidelines. J Med Syst.

[42] Georgsson M, Staggers N (2016). An evaluation of patients' experienced usability of a diabetes mHealth system using a multi-method approach. J Biomed Inform.

[43] Ichikawa D, Kashiyama M, Ueno T (2017). Tamper-Resistant Mobile Health Using Blockchain Technology. JMIR Mhealth Uhealth.

[44] Motohashi T, Hirano T, Okumura K, Kashiyama M, Ichikawa D, Ueno T (2019). Secure and Scalable mHealth Data Management Using Blockchain Combined With Client Hashchain: System Design and Validation. J Med Internet Res.

[45] Dimitrov DV (2019). Blockchain Applications for Healthcare Data Management. Healthc Inform Res.

[46] Coldebella B, Armfield NR, Bambling M, Hansen J, Edirippulige S (2018). The use of telemedicine for delivering healthcare to bariatric surgery patients: A literature review. J Telemed Telecare.

[47] Cunningham SG, Brillante M, Allardice B, Conway N, McAlpine RR, Wake DJ (2019). My Diabetes My Way: supporting online diabetes self-management: progress and analysis from 2016. Biomed Eng Online.

[48] Moon EW, Tan NC, Allen JC, Jafar TH (2019). The Use of Wireless, Smartphone App-Assisted Home Blood Pressure Monitoring Among Hypertensive Patients in Singapore: Pilot Randomized Controlled Trial. JMIR Mhealth Uhealth.

[49] Peres R, Muller E, Mahajan V (2010). Innovation diffusion and new product growth models: A critical review and research directions. International Journal of Research in Marketing.

[50] Rogers E (2003). Diffusion of Innovations, 5th Edition.

[51] Carter A, Liddle J, Hall W, Chenery H (2015). Mobile Phones in Research and Treatment: Ethical Guidelines and Future Directions. JMIR Mhealth Uhealth.

[52] Nebeker C, Lagare T, Takemoto M, Lewars B, Crist K, Bloss CS, Kerr J (2016). Engaging research participants to inform the ethical conduct of mobile imaging, pervasive sensing, and location tracking research. Transl Behav Med.

[53] Radhakrishnan S, Erbis S, Isaacs JA, Kamarthi S (2017). Novel keyword co-occurrence network-based methods to foster systematic reviews of scientific literature. PLoS ONE.

