# Supplementary figures and images for: Determining the Intellectual Structure and Academic Trends of Smart Home Health Care Research: Coword and Topic Analyses
Source: J Med Internet Res. 2021 Jan 21;23(1):e19625. doi: 10.2196/19625 (PMC7862004; doi:10.2196/19625)

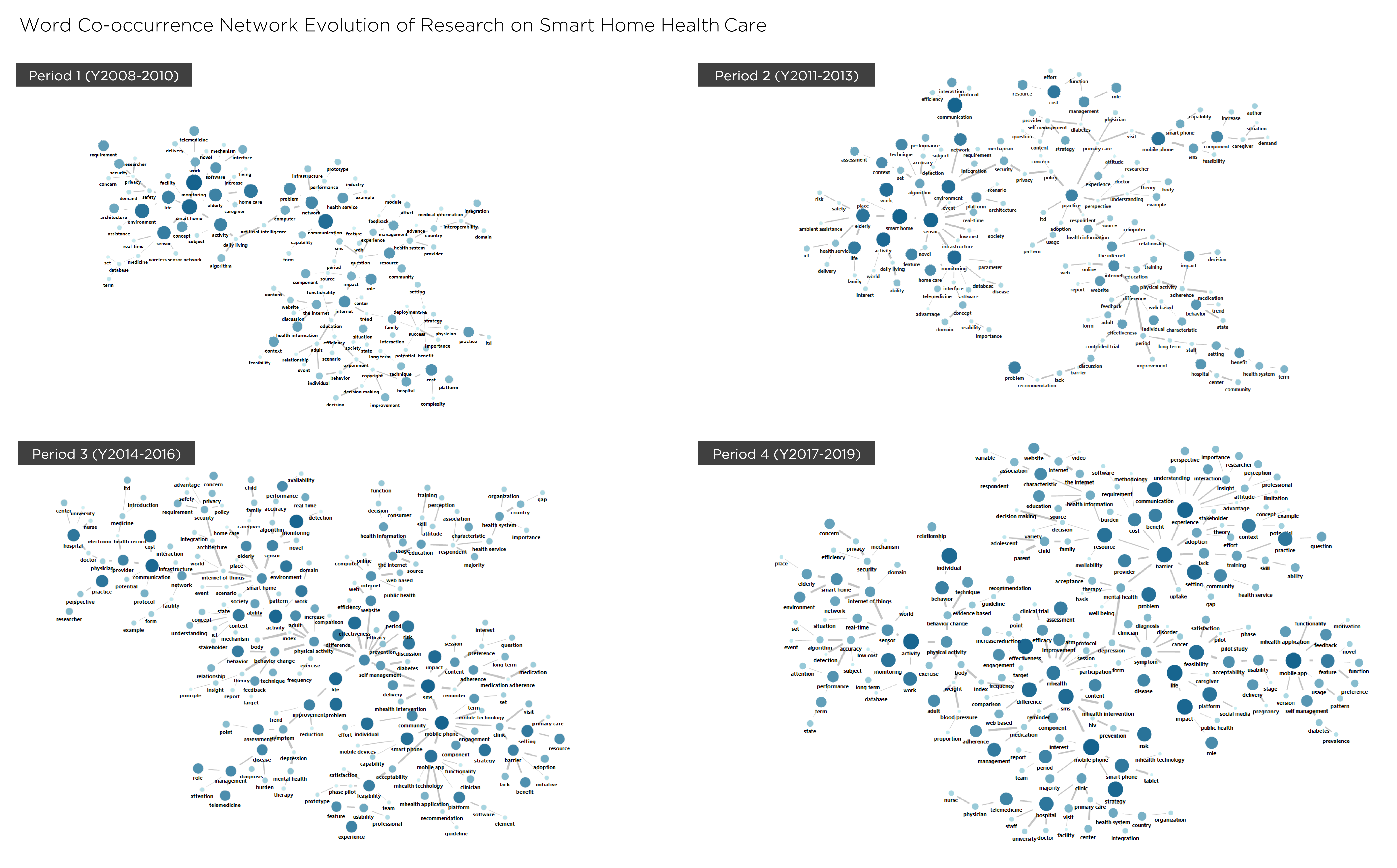

Supplement: Multimedia Appendix 1 [file jmir_v23i1e19625_app1.png]

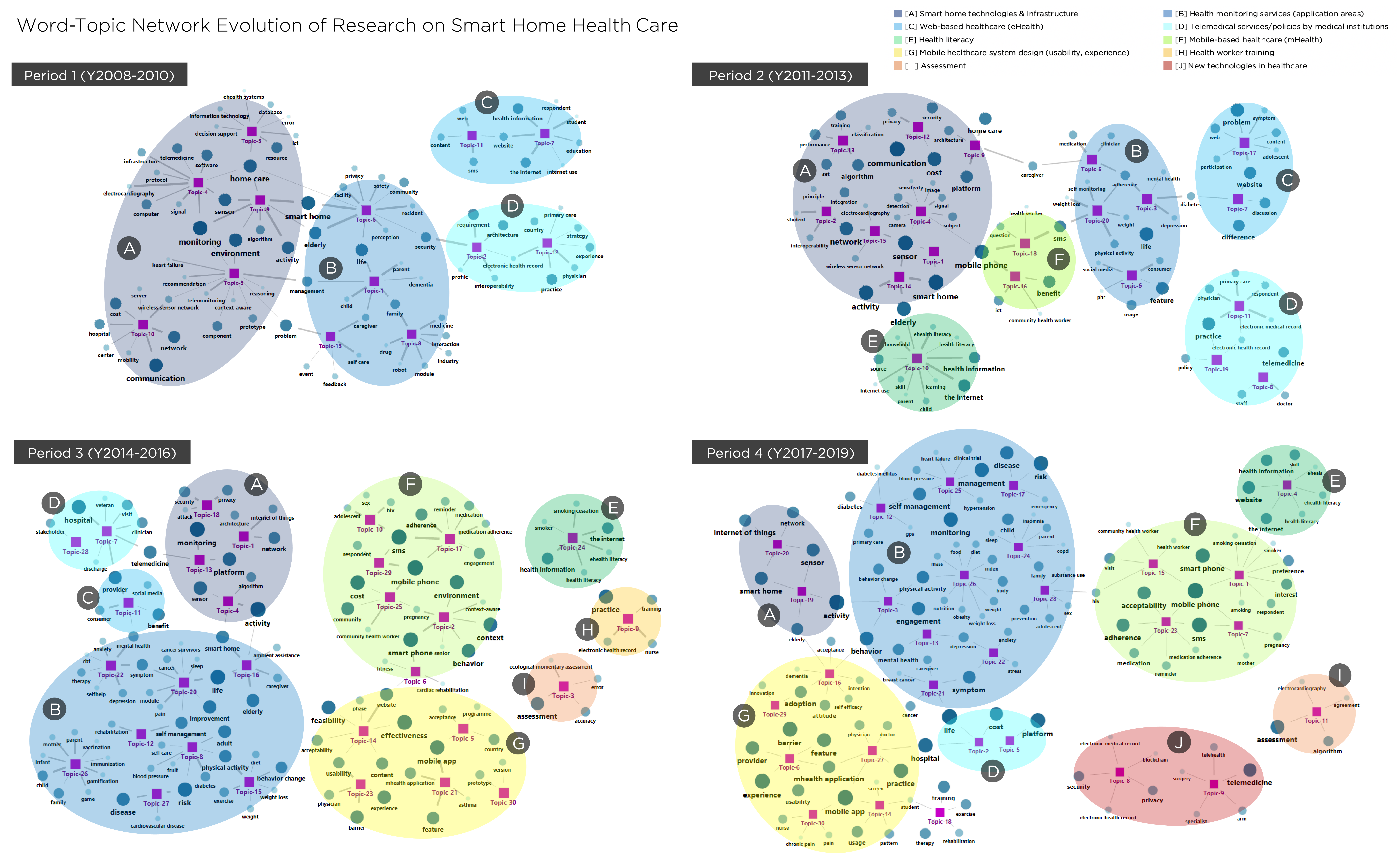

Supplement: Multimedia Appendix 3 [file jmir_v23i1e19625_app3.png]
