# Supplementary material for: Determining the Intellectual Structure and Academic Trends of Smart Home Health Care Research: Coword and Topic Analyses
Source: J Med Internet Res. 2021 Jan 21;23(1):e19625. doi: 10.2196/19625 (PMC7862004; doi:10.2196/19625)
Supplement: Multimedia Appendix 2 [file jmir_v23i1e19625_app2.docx]

**Multimedia Appendix 2.** The evolution of the position of the top 40 keywords by degree centrality in word co-occurrence networks.
